# Alexithymia Is Related to the Need for More Emotional Intensity to Identify Static Fearful Facial Expressions

**DOI:** 10.3389/fpsyg.2018.00929

**Published:** 2018-06-11

**Authors:** Francesca Starita, Khatereh Borhani, Caterina Bertini, Cristina Scarpazza

**Affiliations:** ^1^Department of Psychology, Center for Studies and Research in Cognitive Neuroscience, University of Bologna, Bologna, Italy; ^2^Department of General Psychology, University of Padua, Padua, Italy

**Keywords:** alexithymia, emotional facial expressions, morphing, dynamic facial expressions, fear

## Abstract

Individuals with high levels of alexithymia, a personality trait marked by difficulties in identifying and describing feelings and an externally oriented style of thinking, appear to require more time to accurately recognize intense emotional facial expressions (EFEs). However, in everyday life, EFEs are displayed at different levels of intensity and individuals with high alexithymia may also need more emotional intensity to identify EFEs. Nevertheless, the impact of alexithymia on the identification of EFEs, which vary in emotional intensity, has largely been neglected. To address this, two experiments were conducted in which participants with low (LA) and high (HA) levels of alexithymia were assessed in their ability to identify static (Experiment 1) and dynamic (Experiment 2) morphed faces ranging from neutral to intense EFEs. Results showed that HA needed more emotional intensity than LA to identify static fearful – but not happy or disgusted – faces. On the contrary, no evidence was found that alexithymia affected the identification of dynamic EFEs. These results extend current literature suggesting that alexithymia is related to the need for more perceptual information to identify static fearful EFEs.

## Introduction

The identification of emotional facial expressions (EFEs) is fundamental for social interaction and survival of the individual (Adolphs, 2002). For example, being able to correctly recognize a fearful or a happy facial expression is a crucial adaptive mechanism to infer others’ intentions and anticipate their behavior. Research has shown that this ability is affected not only by clinical conditions such as depression and anxiety (Demenescu et al., 2010) or schizophrenia (Kohler et al., 2009) but also by subclinical differences in the ability to process emotional stimuli, such as alexithymia (Grynberg et al., 2012).

Alexithymia is a personality trait characterized by difficulties in identifying and describing feelings and discriminating between feelings and bodily sensations of emotional arousal, which accompany them (Sifneos, 1973; Taylor et al., 1991). Previous research found alexithymia to be related to worse performance in EFE recognition (Lane et al., 1996; Jessimer and Markham, 1997). Specifically, previous literature mainly manipulated stimulus presentation time, showing that the difficulty in EFE identification was evident when stimuli were presented under temporal constraints but not when stimulus exposure time was extended (for a review see Grynberg et al., 2012). For example, when EFEs were presented for 66 or 100 ms, level of alexithymia was negatively correlated with labeling sensitivity of angry EFEs and marginally negatively correlated with labeling sensitivity of fearful and happy EFEs (Ihme et al., 2014a). On the contrary, no such correlations were found when the same EFEs were presented for 1 or 3 s (Pandey and Mandal, 1997; Ihme et al., 2014b). The implications of these results appear twofold. Firstly, alexithymia may be associated to the need for more time to accurately recognize EFEs. Secondly, the difficulties of alexithymic individuals in EFEs identification appear evident only under certain experimental conditions.

Despite growing evidence on the impact of alexithymia in the identification of EFEs, previous research has focused on the response to intense static EFEs. Nevertheless, these are rarely encountered in everyday life and individuals are faced with the challenge of identifying dynamic changes in emotional expression often displayed at varying degrees of intensity (Sarkheil et al., 2013). In fact, alexithymia may be hypothesized to be related not only to the need for more time but also for more perceptual information to identify EFEs, as previously hypothesized in Grynberg et al. (2012). Therefore, manipulating the intensity of EFEs using both static and dynamic stimuli would enable the extension of current literature on the impact of alexithymia on EFE identification by testing whether or not individuals with alexithymia need more emotional intensity to identify EFEs. Indeed, in the broader literature of emotion processing, the manipulation of emotional intensity can be crucial to uncover impairments in EFE recognition, which are not evident when using intense EFEs (e.g., Willis et al., 2014), making emotion recognition tasks more sensitive to subtle differences in identification (Calder et al., 1996; Wells et al., 2016).

Regarding the issue of intensity in static EFEs, two studies exist that used morphed faces to understand the impact of alexithymia in the identification of static EFEs varying in emotional intensity. Nevertheless, they have the limitations of focusing mainly on alexithymia within the autistic population, reporting contrasting results. Specifically, the first study found alexithymia to be related to less precision, expressed as higher attribution threshold, in the identification of EFEs both in the autistic and control group (Cook et al., 2013). On the contrary, the second study found high levels of alexithymia to be related to reduced accuracy in identifying EFEs at low emotional intensity in the autistic but not in the control group (Ketelaars et al., 2016) raising the possibility that autism *per se* may represent a confounding factor contributing to the results. Given the inconsistency of results, it appears that further research is needed in order to understand the role of emotional intensity in the relationship between alexithymia and EFE identification. In addition, no study has investigated the impact of alexithymia in the identification of dynamic EFEs varying in emotional intensity. Nevertheless, static and dynamic faces appear to convey partially different types of information. Besides being more ecologically valid, dynamic stimuli convey additional temporal information regarding the change of emotional intensity over time (Kamachi et al., 2001), which is not available in static stimuli. This seems to contribute to enhanced perceived intensity of dynamic EFEs (Yoshikawa and Sato, 2008) and has been suggested to facilitate their identification (Sarkheil et al., 2013). In fact, neuroimaging studies have shown that recognizing dynamic as opposed to static morphed EFEs appears not only to enhance the activation of areas involved in affective processing, including the amygdala and fusiform gyrus (LaBar et al., 2003; Trautmann et al., 2009), but also to activate additional brain areas involved in motion processing, including pre- and post-central gyrus, known for sensory-motor integration of motion-related information (Sarkheil et al., 2013).

Given the current literature, the aim of the present study was to investigate the impact of emotional intensity in the relationship between alexithymia and EFE identification when presenting both static and dynamic EFEs. To this end, two experiments were conducted in which participants with low (LA) and high (HA) levels of alexithymia were tested in their ability to identify static (Experiment 1) or dynamic (Experiment 2) morphed EFEs, which ranged from neutral to intense emotional expression.

In both experiments presentation of happy, fearful and disgusted EFEs was chosen for several theoretical reasons. Firstly, both positively and negatively valenced emotions were included to understand if the effect of alexithymia may be valence or emotion related. Secondly, with regards to fear and disgust, these were included because neuroimaging and lesion studies indicate that identification of fearful and disgusted EFEs is related to functional and structural integrity of a circumscribed set of brain areas (Adolphs, 2002). Specifically, the amygdala appears a crucial structure in recognition of fearful EFEs (Adolphs, 2002; Fusar-Poli et al., 2009) and lesion of this structure impairs their recognition (Adolphs et al., 1994, 1999), while the insula appears crucially involved in the recognition of disgusted EFEs (Adolphs, 2002; Fusar-Poli et al., 2009). Regarding alexithymia, aberrant activation of amygdala (e.g., Kugel et al., 2008; Pouga et al., 2010; Wingbermühle et al., 2012; Moriguchi and Komaki, 2013; Jongen et al., 2014) and insula (e.g., Karlsson et al., 2008; Heinzel et al., 2010; Reker et al., 2010) have been found among the neural correlates underlying this condition (for a meta-analysis see van der Velde et al., 2013).

In Experiment 1, compared to LA, HA were hypothesized to need more emotional intensity to identify the emotion expressed by EFEs. On the contrary, in Experiment 2, the additional information inherent to dynamic – as opposed to static – EFEs might facilitate the task, enabling HA to overcome their difficulties. Therefore, in Experiment 2, differences in the emotional intensity needed by HA and LA to identify EFEs may or may not be evident.

## Experiment 1

**FIGURE 1:**
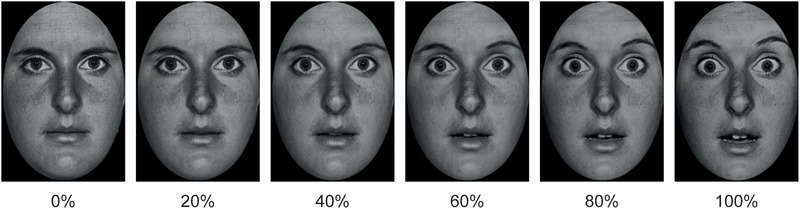
Example of morphed pictures of fearful facial expressions used as EFEs ranging from 0 to 100% emotional intensity.

Participants were presented with pictures of static happy, disgusted and fearful EFEs. The emotion in each EFE could be expressed at 6 levels of emotional intensity: 0, 20, 40, 60, 80, and 100% (**Figure 1**). Participants were required to identify the emotion expressed by the EFE, by making a forced choice button press. In order to test differences between LA and HA, for each participant, expression identification rate for each EFE was calculated at each intensity level. Then, expression identification rates were fit to a psychometric function to calculate the percentage of emotional intensity at which participants had equal probability to identify the facial expression as neutral or emotional, i.e., point of subjective equality (PSE). Compared to LA, HA were hypothesized to need more emotional intensity to identify the presence of the emotional expression in the face, hence showing higher PSE.

### Methods

#### Participants

The study was designed and conducted in accordance with the ethical principles of the World Medical Association Declaration of Helsinki and the institutional guidelines of the University of Bologna and was approved by the Ethics Committee of the Department of Psychology. All participants gave informed written consent to participation after being informed about the procedure of the study.

Three-hundred university students completed the 20-item Toronto Alexithymia Scale (TAS-20; Taylor et al., 2003). Depending on the score, students were classified as LA (TAS-20 ≤ 36) or HA (TAS-20 ≥ 61) (Franz et al., 2004) and were then randomly contacted to participate in the study. Once in the laboratory, the alexithymia module of the structured interview for the Diagnostic Criteria for Psychosomatic Research (DCPR; Mangelli et al., 2006) was administered to increase reliability of screening and confirm TAS-20 classification. Participants with discordant classification on the two measures did not complete the task (*n* = 1). Due to the high co-occurrence of alexithymia and depression (Li et al., 2015), participants completed the Beck Depression Inventory (Beck et al., 1961) and did not complete the experimental task if their score was higher than the cut-off for severe depression (i.e., 28, *n* = 1). All participants had equivalent educational backgrounds and were students at the University of Bologna.

Forty volunteers with no history of major medical, neurological or psychiatric disorders completed the study: 20 LA (6 males; TAS-20 *M* = 30.25, *SD* = 4.12; age *M* = 24.55, *SD* = 2.98 years); 20 HA (6 males; TAS-20 *M* = 63.37, *SD* = 2.25; age *M* = 23.03, *SD* = 2.32 years). *A priori* targets for sample size and data collection stopping rule were based on sample and effect sizes reported in the literature on alexithymia and EFE identification (sample size of an average of 38 participants in total as indicated in a recent review (Grynberg et al., 2012)).

#### Independent Measure

Stimuli consisted of black and white photographs of 20 actors (10 males) with each actor depicting 3 EFEs, respectively of happiness, disgust and fear. Half of the pictures were taken from the Karolinska Directed Emotional Faces database (Lundqvist et al., 1998) and half from the Pictures of Facial Affect database (Ekman and Friesen, 1976). Pictures were trimmed to fit an ellipse in order to uniform them and remove distracting features from the face, such as hair or ears and non-facial contours. Each emotional facial expression was then morphed with the neutral facial expression of the corresponding identity using (Abrosoft FantaMorph, 2009) in order to create stimuli of 20% increments of emotional intensity ranging from 0 to 100% emotional intensity. This resulted in a total of 360 stimuli (20 cm × 13 cm size), i.e., 20 actors expressing 3 emotions with 6 degrees of intensity (0, 20, 40, 60, 80, and 100%; **Figure 1**).

#### Procedure

The experiment took place in a sound attenuated room with dimmed light. Participants sat in a relaxed position on a comfortable chair in front of a computer monitor (17″, 60 Hz refresh rate) used for stimuli presentation at 57 cm distance. Each trial started with the presentation of a fixation cross (500 ms) in the center of the screen followed by the stimulus (100 ms) and subsequently a black screen (3000 ms) during which participants could provide the answer by pressing a key. The experiment consisted of 360 randomized trials divided in two blocks of 180 trials so that participants could rest if desired. Stimulus presentation time was chosen based on previous literature on EFEs recognition, indicating 100 ms as a sufficiently long presentation time to identify EFEs reliably above chance level and without incurring in ceiling effects (Calvo and Lundqvist, 2008; Calvo and Marrero, 2009).

Participants were instructed that at each trial a face would briefly appear on the screen and their task would be to identify the emotion expressed by the face by pressing one of four keys with their index and middle finger of either hand. These were labeled “N” for neutral (i.e., Italian = “neutro”), “F” for happiness (i.e., Italian = “felicità”), “P” for fear (i.e., Italian = “paura”) and “D” for disgust (i.e., Italian = “disgusto”). Before beginning the task, participants familiarized with the position of keys by having the experimenter calling out loud in random order the keys and participants pressing them until they felt confident they could press them correctly while fixating the screen. The order of keys was counterbalanced among participants.

#### Dependent Measure

**FIGURE 2:**
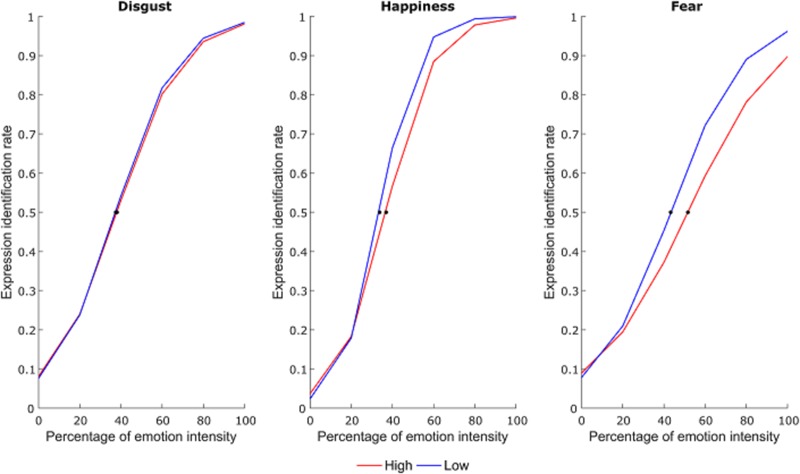
Average psychometric function for each emotional expression as a function of group. The dots represent the point of subjective equivalence (PSE).

Correct responses for each emotional facial expression were used to calculate the mean expression identification rate at each intensity level. Then, for each subject, expression identification rates for each emotional facial expression were fit to a psychometric function using a generalized linear model with a binomial distribution in MATLAB software (MathWorks, Natick, MA, United States) (Nakajima et al., 2017). The point of subjective equality (PSE) was then calculated and used for statistical analysis. This represented the percentage of emotional intensity at which subjects had equal probability to identify the facial expression as neutral or emotional (**Figure 2**).

### Results and Discussion

**FIGURE 3:**
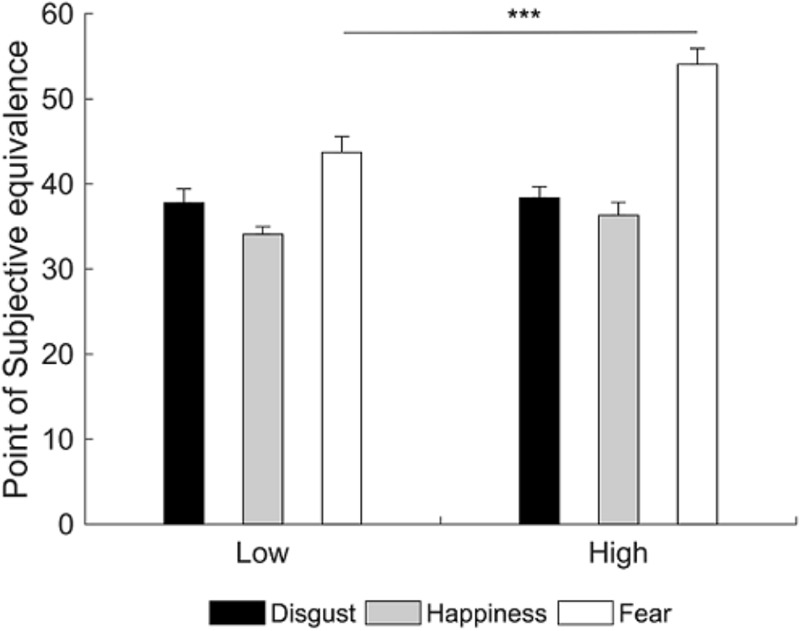
Mean point of subjective equivalence (PSE) for each emotional facial expression as a function of group. Participants with high alexithymia have higher PSE than those with low alexithymia for the fearful facial expression, indicating they need more emotional intensity to identify fearful facial expressions. Error bars represent standard errors. Significant differences are indicated as follows: ^∗∗∗^*p* < 0.001.

A 3×2 repeated measures analysis of variance (RM ANOVA; emotion: happiness, disgust, fear; group: LA, HA) on PSE scores showed a significant main effect of group [*F*(1,38) = 5.38, *p* = 0.026, $\eta_{p}^{2}$ = 0.12] and emotion [*F*(2,76) = 35.75, *p* < 0.001, $\eta_{p}^{2}$ = 0.48]. More importantly, there was a group by emotion interaction [*F*(2,76) = 4.69, *p* = 0.012, $\eta_{p}^{2}$ = 0.11]. Newman-Keuls *post hoc* test shows that HA had higher PSE compared to LA only for the fearful emotional facial expression (fear: *p* < 0.001, *M*_HA_ = 54.05, *M*_LA_ = 43.71; disgust: *p* = 0.832, *M*_HA_ = 38.36, *M*_LA_ = 37.78; happiness: *p* = 0.415, *M*_HA_ = 36.32, *M*_LA_ = 34.08). Therefore, HA need more emotional intensity to identify fearful facial expressions compared to LA (**Figure 3**).

In summary, results showed that HA required more emotional intensity to identify the presence of fear expression in the face compared to LA. Crucially, while previous studies showed that HA need more time to identify EFEs as efficiently as LA (Grynberg et al., 2012), the present study extends the current literature suggesting that HA also need more perceptual information, specifically to identify fearful EFEs.

## Experiment 2

**FIGURE 4:**
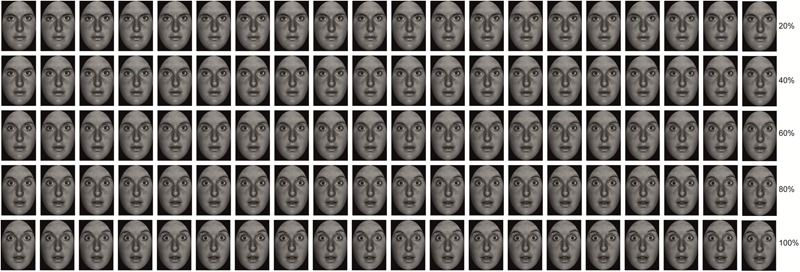
Example of morphed pictures of fearful facial expressions used to create the dynamic EFEs ranging from 0 to 100% emotional intensity.

Participants were presented with videos of dynamic happy, disgusted and fearful EFEs, which started at 0% emotional intensity and terminated at 100% emotional intensity (**Figure 4**). Participants were required to identify the emotion expressed by the EFE, by making a forced choice button press, which would also terminate video presentation. Participants responded as soon as they recognized the emotion, without necessarily waiting for termination of the video. In order to test differences between LA and HA, accuracy and reaction times (RTs) for accurate responses were calculated. Here, RTs also represented the percentage of emotional intensity at which participants identified the emotion displayed by the face. Therefore, differences in RTs indicated differences in the percentage of emotional intensity needed to identify the emotion expressed by the EFE. Contrary to Experiment 1, here, differences in the emotional intensity needed by HA and LA to identify EFEs may or may not be evident, given that dynamic EFEs may be easier to be identified than static ones.

### Methods

#### Participants

Recruitment of participants followed the same procedure as Experiment 1. Two participants did not take part to the experimental task because their TAS-20 classification was not confirmed by their DCPR score. No participant reported a severe level of depression on the BDI.

Forty volunteers with no history of major medical, neurological or psychiatric disorders completed the study: 20 LA participants (8 males; TAS-20 *M* = 31.29, *SD* = 3.23; age *M* = 22.89 years, *SD* = 2.00 years) and 20 HA participants (8 males; TAS-20 *M* = 64.84, *SD* = 4.14; age *M* = 22.84 years, *SD* = 1.93 years).

#### Independent Measure

Stimuli consisted of black and white photographs of 10 actors (5 men) with each actor depicting 3 EFEs, respectively of happiness, disgust and fear. Pictures were chosen from the Pictures of Facial Affect database (Ekman and Friesen, 1976) and trimmed to fit an ellipse in order to uniform stimuli and remove distracting features from the face such as hair or ears and non-facial contours. Each emotional facial expression was then morphed with the neutral facial expression of the corresponding identity using (Abrosoft FantaMorph, 2009) in order to create videos of 1% increments of emotional intensity ranging from 0 to 100% of emotional intensity. Each increment lasted 1 s, resulting in a video with a total duration of 100 s (**Figure 4**). This resulted in a total of 30 stimuli (20 cm × 13 cm size), i.e., 10 actors expressing 3 emotions.

#### Procedure

The experiment took place in a sound attenuated room with dimmed light. Participants sat in a relaxed position on a comfortable chair in front of a computer monitor (17″, 60 Hz refresh rate) used for stimuli presentation at a distance of 57 cm. The experiment consisted of 30 randomized trials, each showing a dynamic facial expression changing from neutral to fear, happiness or disgust. Each trial started with the presentation of a fixation cross (3000 ms) in the center of the screen followed by the presentation of the dynamic stimulus with the duration of 100 s.

Participants were instructed that at each trial a video of a face ranging from neutral to emotional would appear on the screen and their task would be to press one of three keys (D, J, or K) as soon as they recognized the emotion expressed by the face, without having to wait for termination of the video. The keys were labeled “F” for happiness (i.e., Italian = “felicità”), “D” for disgust (i.e., Italian = “disgusto”) and “P” for fear (i.e., Italian = “paura”). Participants used the index and middle fingers of the right hand and the index finger of the left hand to press the keys. The order of keys was counterbalanced between participants. Key press terminated video presentation allowing the task to proceed to the next trial.

#### Dependent Measure

Accuracy (i.e., percentage of correct response) and RTs for accurate responses were calculated. It should be noted that RTs also represented the percentage of emotional intensity at which participants identified the emotion displayed by the face. For example, an average RT of 3000 ms indicated that, on average, a participant correctly identified the emotion displayed by the face when this was expressed at 30% emotional intensity. Therefore, differences in RTs indicated differences in the percentage of emotional intensity needed to identify the emotion expressed by the EFE.

### Results and Discussion

The 3×2 RM ANOVA (emotion: happiness, disgust, fear; group: LA, HA) on accuracy revealed a significant main effect of emotion [*F*(2,76) = 13.83; *p* < 0.001; $\eta_{p}^{2}$ = 0.27]. Newman-Keuls *post hoc* test showed that participants were most accurate in identifying happiness (*M* = 96.05%) than fear (*p* = 0.003; *M* = 90.75%) and disgust (*p* < 0.001; *M* = 86.84%) and were more accurate in identifying fear than disgust (*p* = 0.029). Results showed no significant main effect or interaction with the factor group (all *p*-values ≥ 0.669) indicating that the two groups exhibited comparable accuracy in identifying the emotion expressed by dynamic faces.

Similarly the 3×2 RM ANOVA (emotion: happiness, disgust, fear; group: LA, HA) on RTs revealed a significant main effect of emotion [*F*(2,76) = 78.22; *p* < 0.001; $\eta_{p}^{2}$ = 0.67]. Newman-Keuls *post hoc* test showed that participants were fastest in identifying happiness (*M* = 24770 ms) than fear (*p* < 0.001; *M* = 32623 ms) and disgust (*p* < 0.001; *M* = 36549 ms) and were more accurate in identifying fear than disgust (*p* < 0.001). Results showed no significant main effect or interaction with the factor group (all *p*-values ≥ 0.142) indicating that the two groups required comparable time to identify the emotion expressed by dynamic faces. Because RTs also represent the percentage at which participants recognize the emotion, these results also show that the groups required comparable amount of emotional intensity to identify the emotion expressed by the face.

Contrary to Experiment 1, results of Experiment 2 show no significant difference between LA and HA in accuracy and RTs when identifying the emotion expressed by dynamic morphed faces.

## General Discussion

The aim of the present study was to investigate the role of alexithymia in identifying the emotional expression of static and dynamic EFEs ranging from neutral to intense emotional expression, in order to test whether or not HA need more emotional intensity to identify EFEs. In fact, previous studies have focused on manipulating presentation time of intense static EFEs, revealing that HA need more time to identify EFEs, compared to LA (Grynberg et al., 2012). Here, instead, we manipulated emotional intensity of static and dynamic EFEs. Under these conditions we showed that HA need more emotional intensity to identify static fearful EFEs, compared to LA. Nevertheless, when the groups were faced by dynamic EFEs, no significant difference was found in performance, with groups requiring comparable amount of emotional intensity to identify the EFEs.

In Experiment 1, the difficulty in processing fearful EFEs is in line with previous literature, which found a difficulty of alexithymic individuals in fear processing not only limited to EFEs labeling (Jessimer and Markham, 1997; Lane et al., 2000; Montebarocci et al., 2010) but also across a broad range of stimuli, tasks and dependent measures. For example, compared to LA, HA rate the expression of fearful but not other EFEs as less intense (Prkachin et al., 2009). In addition, HA show impairment in embodied aspects of fearful stimuli processing. This is evidenced by reduced rapid facial mimicry in response to static fearful faces (Sonnby-Borgstrom, 2009; Scarpazza et al., 2018), failure to show enhanced perception of tactile stimuli delivered to their face while observing a fearful – as opposed to happy or neutral – face being simultaneously touched (Scarpazza et al., 2014, 2015) and reduced skin conductance response when viewing a conditioned stimulus predictive of a shock during classical fear conditioning (Starita et al., 2016). Finally, HA show impairments in processing fearful stimuli also when examining their electrophysiological responses. Compared to LA, HA fail to show enhanced amplitude of the N190 event related potential, during visual encoding of fearful – as opposed to happy or neutral – body postures (Borhani et al., 2016). This general difficulty in fear processing has been interpreted in light of the decreased activation of the amygdala observed in alexithymia in response to the presentation of EFEs (Kugel et al., 2008; Jongen et al., 2014), in particular fearful ones (Pouga et al., 2010), and negative emotional stimuli (Wingbermühle et al., 2012; van der Velde et al., 2013), such as observing a painful stimulation being delivered to someone’s hand (Moriguchi and Komaki, 2013). Although involved in processing EFEs in general (Fusar-Poli et al., 2009), the amygdala appears a crucial structure in processing fearful EFEs (Adolphs et al., 1994, 1999). Therefore, it is possible that a reduced response in the amygdala in HA may underlie the present results, though future studies using neuroimaging techniques should be conducted to test this hypothesis.

In contrast to the difference found in response to fearful EFEs, no difference between the groups was found when identifying happy or disgusted facial expressions. In this regard, previous behavioral studies on EFEs processing have reported mixed results. For example, in Prkachin et al. (2009), though HA showed reduced sensitivity for matching sad, angry and fearful faces to the corresponding target EFE, they showed no significant difference from LA when matching happy, disgusted or surprised EFEs; in addition, they were able to recognize all EFEs during a non-speeded task and rated the intensity of happy and disgusted EFEs similarly to LA. On the contrary, other labeling studies found that alexithymia was related to a global deficit to recognize EFEs, including happiness and disgust (Jessimer and Markham, 1997; Lane et al., 2000; Montebarocci et al., 2010). Given the contrasting results, alexithymia may affect processing of happy and disgusted EFEs depending on the experimental conditions. Specifically, here results seem to suggest that while HA require more emotional intensity to identify static fearful EFEs, they may not have such need in the identification of happy and disgusted EFEs.

Contrary to Experiment 1, when dynamic morphed faces were presented in Experiment 2, no difference was found between the two groups in EFE recognition. This result may be related to the type of information conveyed by dynamic as opposed to static stimuli. Indeed, the intensification of emotional expression over time provides additional structural and configurational information, which is not available in static stimuli (Kamachi et al., 2001) and which seems to contribute to differential processing of the two types of stimuli. For example, dynamic EFEs are perceived as more intense than static ones even when the stimulus emotional intensity is the same (Yoshikawa and Sato, 2008; Rymarczyk et al., 2011; Rymarczyk et al., 2016a,b). In addition, dynamic EFEs trigger stronger facial mimicry compared to static faces (Sato et al., 2008; Rymarczyk et al., 2011, 2016a,b). Finally, recognizing dynamic as opposed to static morphed EFEs activates an extended neural network comprising not only areas involved in affective processing (LaBar et al., 2003; Sato et al., 2004), but also motion processing (Sarkheil et al., 2013). It is possible that the involvement of such additional mechanisms during the identification of dynamic EFEs might have facilitated the task and led to the absence of significant differences in performance between HA and LA. Future studies should investigate this hypothesis and in particular test whether reduced facial mimicry found in HA in response to static fearful EFEs (Sonnby-Borgstrom, 2009; Scarpazza et al., 2018) may be restored by the presentation of dynamic EFEs and be related to improvement in dynamic EFE identification. Additionally, the comparable performance in dynamic EFEs identification between HA and LA highlights the subclinical nature of alexithymia, further supporting the notion that difficulties in EFE identification of HA become evident only under specific task conditions (Grynberg et al., 2012) and may not necessarily be evident in their everyday life.

To conclude, the present study shows that high – as opposed to low - levels of alexithymia are related to the need for more emotional intensity to perceive fear in static EFEs. On the contrary, no significant difference in performance was found when individuals with high and low levels of alexithymia were faced by dynamic EFEs, possibly due to the additional structural and configurational information regarding the change of emotional intensity over time (Kamachi et al., 2001), which may have facilitated emotion identification. Given that partially different brain networks are involved in processing the two types of stimuli, future studies should use neuroimaging techniques to elucidate the neural mechanisms underlying the current behavioral results.

## Availability of Data

The raw data supporting the conclusions of this manuscript will be made available by the authors, without undue reservation, to any qualified researcher.

## Author Contributions

FS, KB, CB, and CS conceived and designed the study and critically revised the manuscript for important intellectual content. FS, KB, and CB acquired and analyzed the data and drafted the manuscript.

## Conflict of Interest Statement

The authors declare that the research was conducted in the absence of any commercial or financial relationships that could be construed as a potential conflict of interest.
